# Evaluation of TyG index and TG/HDL-C ratio in HBeAg negative chronic hepatitis B infected patients

**DOI:** 10.17305/bjbms.2022.7320

**Published:** 2023-01-06

**Authors:** Tülay Ünver Ulusoy, Heval Can Bilek, Hacer Demirköse, Ayşegül Keleş

**Affiliations:** 1 Department of Infectious Diseases and Clinical Microbiology, University of Health Sciences, Diskapi Yildirim Beyazit Training and Research Hospital, Ankara, Turkey; 2 Department of Infectious Diseases and Clinical Microbiology, Ondokuz Mayıs University Faculty of Medicine, Samsun, Turkey; 3 Pursaklar District Health Directorate, Public Health, Ankara, Turkey; 4 Yıldırım Beyazıt University Yenimahalle Training and Research Hospital, Biochemistry, Ankara, Turkey

**Keywords:** TyG index, TG/HDL-C ratio, HBV infection, insulin resistance

## Abstract

Patients with hepatitis B e antigen (HBeAg) negative chronic HBV infection are regularly followed up. This study investigates the presence of insulin resistance and the relationship between hepatosteatosis and insulin resistance in patients with HBeAg negative chronic HBV infection using the TyG index and TG/HDL-C ratio. Patients with HBeAg negative chronic HBV infection who applied to the Infectious Diseases and Clinical Microbiology outpatient clinic between January 2019 and December 2020 were included in the study. Glucose, lipid panel, alanine amino transferase (ALT), acetyl amino transferase (AST), body mass index (BMI), TyG index, TG/HDL-C, and hepatobiliary ultrasonography results were evaluated. The data were compared with the control group consisting of 308 HBsAg negative individuals. The study included 132 patients with a median age of 52 years. There was no significant difference between the patient and control groups regarding age, gender, and BMI. Glucose, total cholesterol, TG, LDL, AST, ALT, TyG index, and TG/HDL-C ratio were significantly higher in patient than in the control group. At the same time, the HDL value was significantly lower in the patients. There was a strong positive correlation between the TG and BMI, and a strong negative correlation between HDL levels and both TyG index and TG/HDL-C ratio. Our findings showed that the TyG index and TG/HDL-C ratio are helpful in the diagnosis of insulin resistance and hepatosteatosis in patients with HBeAg negative chronic HBV infection.

## Introduction

Hepatitis B virus (HBV) infection is a global public health problem. It is estimated that there are more than 250 million HBV-infected patients worldwide, and approximately 600,000 of these people die annually from chronic liver diseases associated with HBV [1]. In the European Association for the Study of the Liver (EASL) 2017 clinical practice guideline on the management of HBV infection, the infection is evaluated in five stages [2]. The third stage of HBV infection was named “hepatitis B e-antigen (HBeAg) negative chronic HBV infection (CHB),” previously known as the “inactive HBsAg carrier state” [2]. In this phase, hepatitis B surface antigen (HBsAg) is positive, HBeAg negative, anti-HBe positive, HBV deoxyribonucleic acid (HBV-DNA) undetectable or <10,000 copies/ml, and alanine aminotransferase (ALT) usually below the threshold value. In addition, there is minimal hepatic necroinflammatory activity and a low level of fibrosis. These patients are less likely to develop cirrhosis or hepatocellular carcinoma (HCC), but they may revert to the next stage, HBeAg-negative chronic hepatitis [2]. This situation increases the likelihood of developing HCC and cirrhosis. Therefore, ALT is monitored every 3 months in the 1^st^ year to detect the inactive phase, and patients are followed every 6–12 months in the following years [3]. In this patient group, insulin resistance is not emphasized in daily practice and insulin, glucose and blood lipid levels are not among the parameters recommended to be followed routinely.

In the presence of insulin resistance, insulin sensitivity of peripheral tissues decreases and hyperinsulinism develops accordingly. Insulin resistance is a common pathophysiological mechanism for metabolic abnormalities, including diabetes mellitus (DM), obesity, and metabolic syndrome [4]. Hyperinsulin in peripheral and portal veins induces *de novo* lipogenesis, changes the oxidation of fatty acids, increases the release of free fatty acids rich in adipose tissue, and causes the accumulation of these products in the liver. The cycle of all these events leads to hepatosteatosis [4]. The gold standard for evaluating insulin resistance is the euglycemic hyperinsulinemic clamp test [5]. However, since this method is time-consuming, costly and difficult to access, more practical tests for detecting insulin resistance are needed. Guerrero-Romero et al. showed that insulin resistance could be detected practically with the triglyceride-glucose (TyG) index [6]. Homeostatic model assessment for insulin resistance (HOMA-IR) or other indices derived from insulin measurement is widely used in daily practice. Still, a growing number of studies indicate that the TyG index and triglyceride (TG) and high-density lipoprotein-C (HDL-C) ratio are also indicators of insulin resistance and which are convenient and practical [6–8].

Hepatosteatosis is a common histopathological finding in CHB patients [9]. However, the relationship between hepatosteatosis and insulin resistance in patients with CHB has not been fully elucidated. This study aims to investigate the presence of insulin resistance and the relationship between hepatosteatosis and insulin resistance in patients with HBeAg negative chronic HBV infection using the TyG index and TG/HDL-C ratio.

## Materials and methods

### Patients and study design

Patients aged 18 years and older with HBeAg negative CHB infection who applied to Yıldırım Beyazıt University Yenimahalle Training and Research Hospital Infectious Diseases and Clinical Microbiology outpatient clinic between January 2019 and December 2020 included. The diagnosis of the disease was made by an Infectious Diseases and Clinical Microbiology physician according to the EASL 2017 guideline [2]. The patients were men and women with HBsAg positive, anti-HBs negative, HBeAg negative, anti-HBe positive, HBV-DNA PCR <10,000 copies/ml, and ALT levels less than twice the reference value.

Patients who received treatment for HBV infection had another known liver disease, were on routine medication, were a consumer of alcohol, and were pregnant were not included in the study. The control group consisted of HBsAg negative male and female volunteers, aged between 18 and 99 years, who applied to the Infectious Diseases and Clinical Microbiology outpatient clinic to determine hepatitis serology without any clinical symptoms and known comorbid diseases.

Demographic, laboratory, and radiological data of the patients were analyzed retrospectively from the hospital medical information system, and other disease and drug use information was obtained from the personal health system of the Ministry of Health [10].

Body mass index (BMI) is calculated by dividing the body weight (kilogram, kg) by the height (centimeter, cm) square. BMI was classified as <18.5: Underweight, 18.5–24.9: Normal weight, 25–29.9: overweight, 30–40: obese, and>40: morbidly obese [11].

TyG index was calculated as (Ln (TG [mg/dL]×- glucose [mg/dL]/2)) [6]. Insulin resistance for the patient and control groups was calculated using the TyG index or the TG/HDL-C ratio. The patient and control groups were divided into groups with a TyG index below or above 8.5 and a TG/HDL-C ratio below or above three. Patients with a TyG index of ≥8.5 and a TG/HDL-C ratio of ≥3 were considered as patients with a high incidence of insulin resistance.

### Laboratory parameters

Serum fasting glucose, total cholesterol, TG, HDL-C, low-density lipoprotein (LDL), acetyl aminotransferase (AST), and ALT tests are studied on the autoanalyzer device of the hospital’s central biochemistry laboratory (Beckman coulter AU 5800, California, USA) following instructions and the manufacturer’s original kits.

### Radiological evaluation

Hepatosteatosis was evaluated with hepatobiliary ultrasonography (USG) reports. A subjective classification including hepatic, periportal, and diaphragmatic echogenicity was used. According to the results, patients were grouped as those without hepatosteatosis and those with Grade 1, Grade 2, and Grade 3 hepatosteatosis.

### Virological evaluations

HBsAg, HBeAg, Anti-HBe, and Anti-HBs markers were studied on the ARCHITECT i2000 system using ARCHITECT chemiluminescent microparticle immunoassay kits (Abbott Park, Wiesbaden - Delkenheim, Germany). HBV-DNA levels were quantitatively analyzed using the HBV QS-RGQ Kit (Qiagen, Germany) and the LightCycler real-time PCR System (Corbett Research Rotor-Gene 6000). HBV-DNA PCR value results were reported as copies/ml.

### Ethical statement

Ethics committee approval was obtained from Yıldırım Beyazıt University Yenimahalle Training and Research Hospital for our single-center study (decision no: 2020-3-8, decision date: 16.12.2020).

### Statistical analysis

Research data were evaluated using SPSS 23.0 statistical package program. The descriptive statistics section presented categorical variables as numbers and percentages, while continuous variables were presented as the median (minimum and maximum values). Conformity of continuous variables to normal distribution was evaluated using visual (histogram and probability graphs) and analytical methods (Kolmogorov–Smirnov/Shapiro–Wilk tests). The Mann-Whitney U test used for comparative analysis between two independent groups. The Kruskal–Wallis test was used for a 3-group comparison that did not fit the normal distribution. Spearman correlation analysis was performed between the TyG index and TG/HDL-C ratio and some parameters in the patient group. The correlation coefficient was categorized as 0–0.25 weak, 0.26–0.50 moderate, 0.51–0.75 strong, and 0.76–1.00 very strong. The Chi-square test was used in the comparison analysis for categorical variables among independent groups. *p* < 0.05 was considered statistically significant.

**Table 1:** Comparison of demographic and laboratory findings of patient and control groups

	**Patient group (*n* ═ 132)**	**Control group (*n* ═ 308)**	***p***
Age, years			
Median (min-max)	52 (27–77)	47 (27–90)	0.165^1^
BMI, kg/m^2^			
Median (min-max)	25.12 (16–44.9)	24.95 (17.7–45)	0.172^1^
Gender, *n* (%)			
Female	69 (52.3)	162 (52.6)	0.950^2^
Male	63 (47.7)	146 (47.4)	
Glucose, mg/dl			
Median (min-max)	92 (74–244)	91.6 (59–230)	**0.001** ^1^
T. cholesterol, mg/dl			
Median (min-max)	196.0 (116.0–304.0)	184.75 (86.0–264.0)	**<0.001** ^1^
TG, mg/dl			
Median (minmax)	107.0 (39.0–488.0)	91.85 (15.0–182.0)	**<0.001** ^1^
HDL, mg/dl			
Median (min-max)	47.0 (29.0–98.0)	49.6 (26.0–99.0)	**0.035** ^1^
LDL, mg/dl			
Median (min-max)	122.5 (57.0–222.0)	114.6 (43.0–186.0)	**0.001** ^1^
AST, IU/L			
Median (min-max)	21.0 (12.0–42.0)	18.9 (10.0–42.0)	**<0.001** ^1^
ALT, IU/L			
Median (min-max)	18.0 (6.0–75.0)	16.5 (5.0–53.0)	**0.008** ^1^
TyG Index			
Median (min-max)	8.52 (7.44–10.33)	8.32 (6.0–10.2)	**<0.001** ^1^
TG/HDL-C Ratio			
Median (min-max)	2.25 (0.49–15.25)	1.86 (0.28–6.71)	**<0.001** ^1^
TyG Index			
<8.5	64 (48.5)	195 (63.3)	**0.004** ^2^
≥8.5	68 (51.5)	113 (36.7)	
TG/HDL-C Ratio	–		
<3	88 (66.7)	260 (84.4)	**<0.001** ^2^
*≥*3	44 (33.3)	48 (15.6)	

^1^Mann–Whitney U Test; ^2^Chi-Square Test, *Column percentage. BMI: Body mass index; TG: Triglyceride; HDL: High-density lipoprotein; LDL: Low-density lipoprotein; AST: Acetyl aminotransferase; ALT: Alanine aminotransferase; TyG: Triglyceride-glucose. Bold entries are significant at <0.05.

## Results

In the patient group, there were 69 (52.3%) women, 63 (47.7%) men, a total of 132 patients, and the median age was 52 years (min.27, max.77). According to BMI, 18 (13.6%) of the patients were underweight, 62 (47%) were normal weight, 45 (34.1%) were overweight, and 7 (5.3%) were obese. There was no hepatosteatosis in 78 (59%) of the patients, Grade 1 hepatosteatosis was found in 36 (27.2%), and Grade 2 hepatosteatosis was found in 18 (13.6%) patients. Patients were divided into two groups, those with and without hepatosteatosis, according to hepatobiliary USG results.

**Figure 1.**
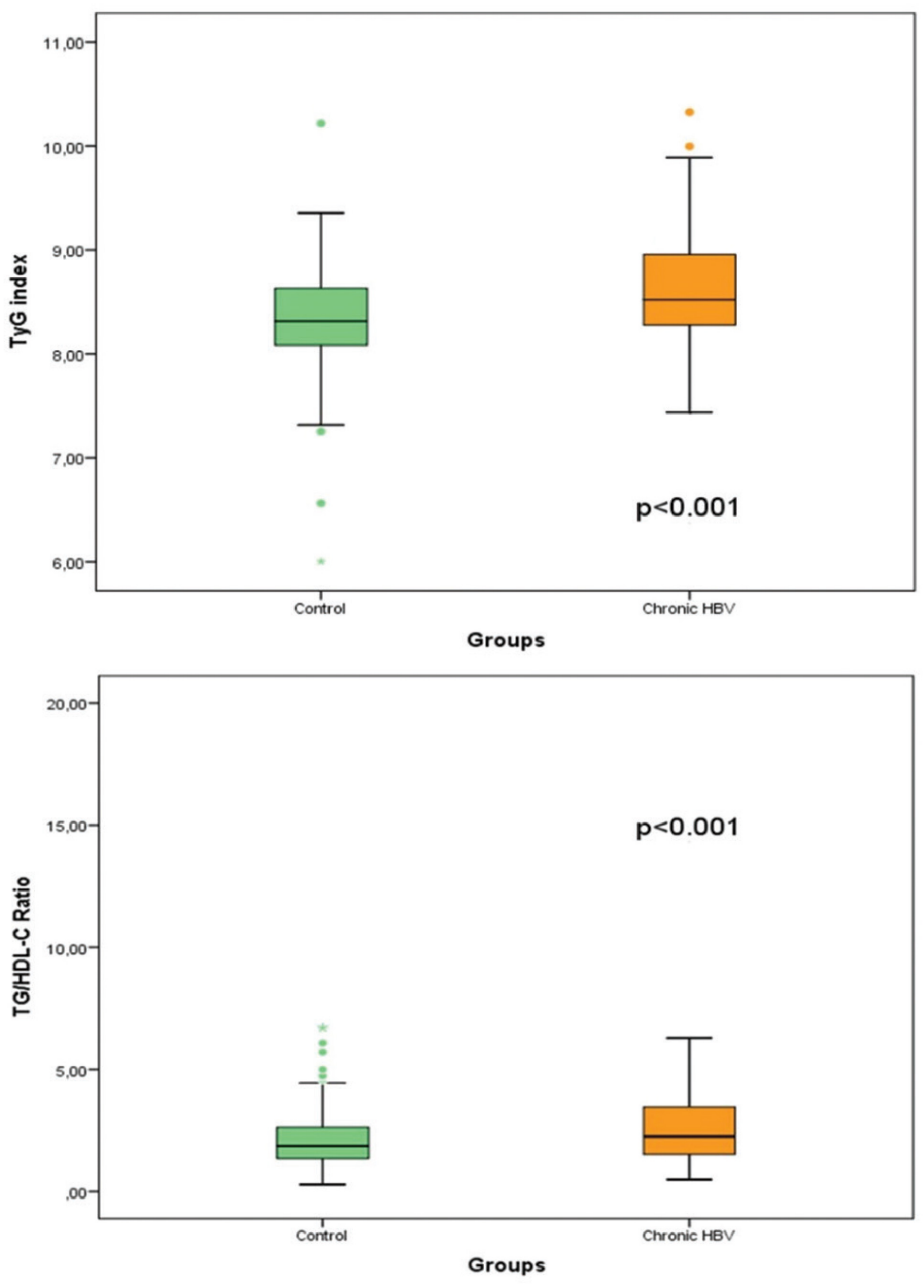
**Comparison of TyG index and TG/HDL-C ratio of patient and control groups.** TyG: Triglyceride-glucose; TG: Triglyceride; HDL-C: High-density lipoprotein cholesterol.

There were a total of 308 cases, 162 (52.6%) women and 146 (47.4% men) men in the control group, and the median age was 47 years (min.27, max.90). According to BMI, 31 (10.1%) of the cases were underweight, 138 (44.8%) were normal-weight, 118 (38.3%) were overweight, 19 (6.1%) were obese, and two patients (0.7%) were morbidly obese. The median BMI of the control group was 24.95 (min.17.7, max.45). There was no significant difference between the patient and control groups regarding age, gender, and BMI (*p* ═ 0.165, *p* ═ 0.950, *p* ═ 0.72) (Table 1). Fasting serum glucose, total cholesterol, TG, LDL, AST, ALT, TyG index, and TG/HDL-C ratio in the patient group were statistically significantly higher than the control group (*p* ═ 0.001, *p* < 0.001, *p* < 0.001, *p* ═ 0.001, *p* < 0.001, *p* ═ 0.008, *p* < 0.001, and *p* < 0.001) (Table 1 and Figure 1). The TyG index ≥8.5 and the TG/HDL-C ratio ≥3 in the patient group were significantly higher (*p* ═ 0.004, *p* < 0.001). At the same time, the HDL value was significantly lower (*p* ═ 0.035).

The relationship between the TyG index and TG/HDL-C ratio and the gender, hepatosteatosis on USG, HBV-DNA levels, and BMI of the patient group was examined. The TyG index was found to be statistically high (*p* ═ 0.030, *p* < 0.001, and *p* ═ 0.018) in males with high BMI and the presence of hepatosteatosis. No significant correlation was found between HBV DNA level and TyG index (*p* ═ 0.724) (Table 2).

**Table 2:** Comparison of TyG index and TG/HDL-C ratio by characteristics in the patient group

	**TyG index Median (min-max)**	***p***	**TG/HDL-C ratio Median (min-max)**	***p***
Gender				
Female	8.47 (7.44–10.33)	**0.030** ^1^	1.75 (0.28–7.06)	**<0.001** ^1^
Male	8.67 (7.56–10.0)		2.26 (0.62–5.25)	
Hepatosteatosis				
No	8.39 (7.44–9.75)	**0.018** ^1^	2.07 (0.49–7.86)	**0.034** ^1^
Yes	8.68 (7.58–10.33)		2.52 (0.66–5.25)	
HBV DNA				
Negative	8.48 (7.44–9.71)	0.724^2^	2.20 (0.66–8.46)	0.882^2^
<5000	8.65 (7.44–10.33)		2.27 (0.49–5.25)	
≥5000	8.46 (7.88–9.89)		2.30 (1.10–7.86)	
BMI				
<18.5	7.65 (7.44–8.98)	**<0.001** ^1^	1.06 (0.81–0.68)	**<0.001** ^1^
18.5–24.9	8.35 (7.44–9.75)		1.82 (0.49–0.85)	
25–29.9	8.79 (7.88–9.68)		2.82 (1.11–0.29)	
>30	9.41 (8.45–10.33)		5.26 (1.67–15.25)	

^1^Mann–Whitney U Test: ^2^Kruskal–Wallis Test; HBV DNA: Hepatitis B virus deoxyribonucleic acid; BMI: Body mass index; TyG: Triglyceride-glucose; TG: Triglyceride; HDL-C: High-density lipoprotein cholesterol. Bold entries are significant at <0.05.

The TG/HDL-C ratio was statistically higher in men, in those with high BMI, and in the presence of hepatosteatosis (*p* < 0.001, *p* < 0.001, *p* ═ 0.034), and it was not significantly associated with HBV-DNA level (*p* ═ 0.882).

**Figure 2.**
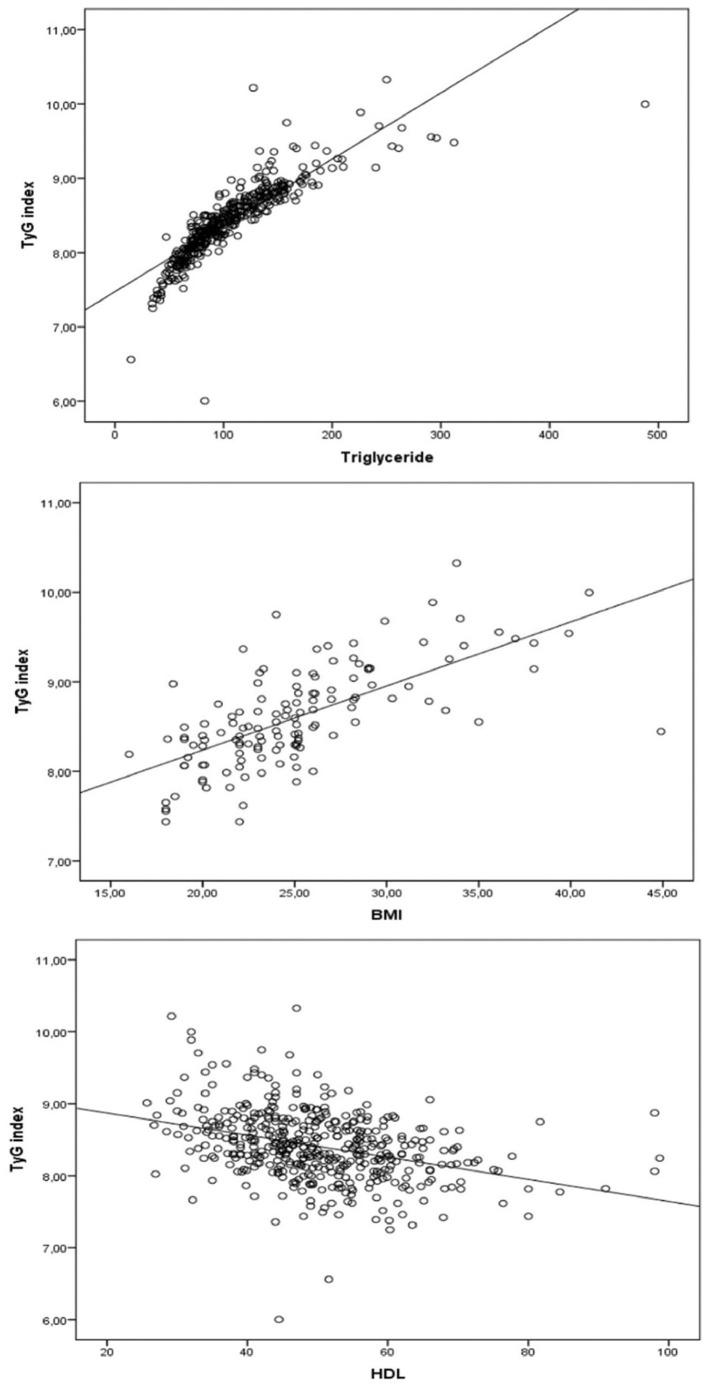
**Evaluation of the relationship between triglyceride, BMI, HDL, and TyG index in the patient group.** BMI: Body mass index; TyG: Triglyceride-glucose; HDL: High-density lipoprotein.

There was no correlation between the TyG index and the age, LDL, AST, and HBV-DNA level of the patient group ([r: 0.115; *p* ═ 0.190], [r: 0.099; *p* ═ 0.257], [r: 0.023; *p* ═ 0.792], and [r: 0.011; *p* ═ 0.920]). There was a weak correlation with total cholesterol (r: 0.217; *p*: 0.012) and ALT (r: 0.180; *p*: 0.039), a moderate positive correlation with glucose (r: 0.469; *p* < 0.001), and there was also a strong positive correlation with TG (r: 0.946; *p* < 0.001) and BMI (r: 0.699; *p* ═ 0.001) (Table 3 and Figure 2).

**Table 3:** Evaluation of the relationship between some parameters and TyG ındex and TG/HDL-C ratio in the patient group

***N* ═ 132**	**TyG r (p)**	**TG/HDL-C r (p)**
Age	0.115 (0.190)	0.025 (0.597)
Glucose, mg/dl	**0.469 (<0.001)**	**0.177 (<0.001)**
BMI	**0.699 (0.001)**	**0.680 (0.001)**
Total cholesterol, mg/dl	**0.217 (0.012)**	0.092 (0.053)
HDL, mg/dl	**−0.541 (<0.001)**	**−0.697 (<0.001)**
LDL, mg/dl	0.099 (0.257)	**0.115 (0.016)**
ALT, IU/L	**0.180 (0.039)**	**0.293 (<0.001)**
AST, IU/L	0.023 (0.792)	**0.146 (0.002)**
HBV DNA, kopya/ml	0.011 (0.920)	0.021 (0.849)

r: Spearman correlation coefficient; BMI: Body mass index; HDL: High-density lipoprotein; LDL: Low-density lipoprotein; ALT: Alanine aminotransferase; AST: Acetyl aminotransferase; HBV DNA: Hepatitis B virus deoxyribonucleic acid; TyG: Triglyceride-glucose; TG: Triglyceride; HDL-C: High-density lipoprotein cholesterol. Bold entries are significant at <0.05.

**Figure 3.**
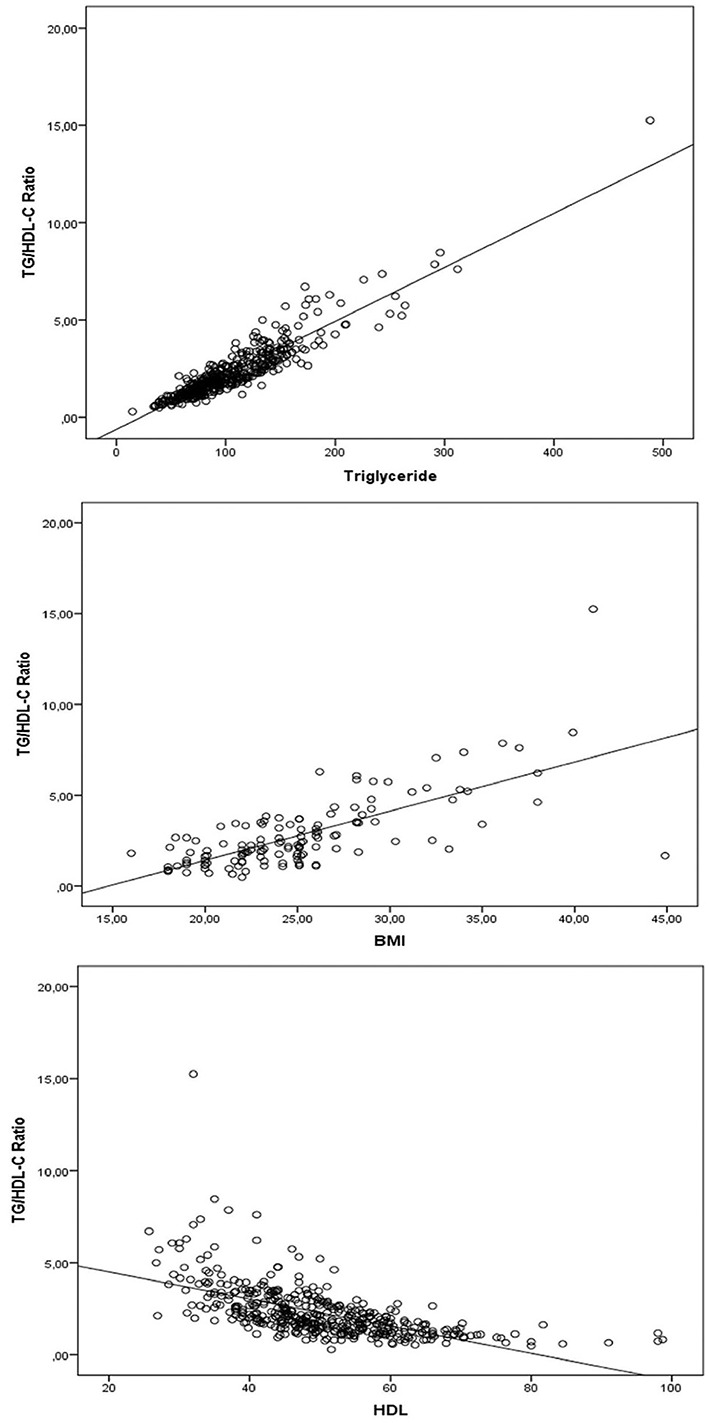
**Evaluation of the relationship between triglyceride, BMI, HDL, and TG/HDL-C ratio in the patient group.** BMI: Body mass index; TyG: Triglyceride-glucose; TG: Triglyceride; HDL-C: High-density lipoprotein cholesterol.

There was no correlation between the TG/HDL-C ratio and the age, total cholesterol, and HBV DNA levels of the patient group ([r: 0.025; *p* ═ 0.597], [r: 0.092; *p* ═ 0.053], [r: 0.021; *p* ═ 0.849]). There was a weak correlation between glucose (r: 0.177; *p* < 0.001), LDL (r: 0.115; *p*: 0.016), and AST (r: 0.146; *p* ═ 0.002, a moderate correlation with ALT (r: 0.293; *p* < 0.001), and a positive high correlation with TG (r: 0.910; *p* < 0.001) and BMI (r: 0.680; *p* ═ 0.001) (Figure 3).

There was a strong negative correlation between HDL level and both TyG index and TG/HDL-C ratio ([r: 0.441; *p* < 0.001), (r: 0.697; *p* < 0.001]).

## Discussion

Our findings showed that the TyG index and TG/HDL-C ratio were significantly higher in patients with HBeAg negative chronic HBV infection compared to the control group. At the same time, a strong positive correlation was found between TG and BMI in the patient group, and a strong negative correlation was found between HDL levels, TyG index and TG/HDL-C ratio. TyG index and TG/HDL-C ratio were found to be statistically higher in men, those with high BMI and the presence of hepatosteatosis. We interpreted these results as a higher risk of developing a metabolic disease related to insulin resistance in the patient group. Insulin resistance causes endothelial dysfunction, vascular resistance, hypertension, inflammation of vascular endothelium, and microvascular damage. While, endothelial dysfunction causes atherosclerosis and hypertension, it also causes peripheral vascular diseases and heart and kidney failures resulting from left ventricular hypertrophy [12]. The TyG index is a reliable indicator of insulin resistance that has been used recently, using plasma triglyceride and glucose parameters [6]. Furthermore, its correlation with HOMA-IR and the euglycemic clamp test has been demonstrated [6, 13]. Studies have shown that the TyG index is associated with an increased risk of DM, hypertension, non-alcoholic fatty liver disease (NAFLD), cardiovascular events, and metabolic syndrome [14–18]. The TG/HDL-C ratio is accepted as an indicator of metabolic syndrome, insulin resistance, and cardiovascular disease [7, 8, 19].

Navarro-González et al. investigated the incidence of cardiovascular and metabolic diseases, including 4820 people without DM, and it was found that the TyG index increased with increasing age [14]. Fan et al. reported that the TG/HDL-C ratio increased with age in a study that included 18.061 people without chronic liver disease who applied to the hospital for routine health check-ups [16]. Our study showed no significant correlation between patient age, TyG index, and TG/HDL-C ratio. This result may be due to the fact that the metabolic effects of the virus in individuals infected with HBV are independent of the age factor. The estrogen hormone is known to protect against cardiovascular diseases, metabolic syndrome and related complications in women [20, 21]. The previous studies reported that males’ TyG index and TG/HDL-C ratio were higher [14, 16]. In our study, these two values were used as markers for insulin resistance and related metabolic complications and were higher in males. We concluded that this result was related to the male gender rather than HBV infection.

In the study of Senoymak and Ozkan factors associated with insulin resistance were investigated in people with HBeAg negative chronic HBV infection and HOMA-IR≥2.5. While the TG/HDL-C ratio was an independent factor in predicting insulin resistance, no significant correlation was found between insulin resistance and HBV-DNA level [18]. In our study, insulin resistance was evaluated with the TyG index and HDL/TG-C ratio, and similarly, no relationship was found between insulin resistance and HBV-DNA level. This result may be due to our study’s inclusion of patients with HBV DNA <10,000 copies/ml viral load. It may also be because the metabolic changes caused by HBV are independent of the viral load level.

High plasma triglyceride levels predispose to coronary artery disease, which is associated with insulin resistance, type 2 DM, and metabolic syndrome [22, 23]. Dyslipidemia is characterized by increased TG, increased LDL, and decreased HDL in diabetic patients and occurs secondary to insulin resistance [23]. Therefore, dyslipidemia, insulin resistance and DM often coexist or trigger each other. Su et al. stated that HDL-C is lower in chronic hepatitis b patients and emphasized the need for follow-up regarding atherosclerotic heart diseases [24]. Sánchez-Íñigo et al., in their study, in which 5014 people without DM and hypertriglyceridemia were evaluated, it was pointed out that the TyG index was higher in normoglycemic people with low HDL and people with a TyG index above 8.45 were at risk for cardiovascular disease [25]. In our study, the TyG index median value of the patients was 8.52, TG LDL levels were higher, and HDL levels were lower. These findings in patients; suggested a higher risk of atherosclerosis due to dyslipidemia.

It has been shown in the previous studies that the possibility of developing insulin resistance and related complications increases in obese patients [26]. Although normal weight patients were in the majority in our study, the TyG index and HDL/TG-C ratio were significantly higher in patients with high BMI and showed a positive correlation with the increase in BMI. This finding indicated that insulin resistance should be considered in obese patients with HBeAg negative chronic HBV infection.

Yu et al. pointed out that the TyG index can be used in individuals at risk for metabolic diseases with liver dysfunction [27]. ALT is an important marker of liver damage and is also used to detect hepatosteatosis [28]. Hepatosteatosis is defined as fat accumulation in the liver exceeding 5% or more of the total liver weight or microscopically more than 5% fat deposits in hepatocytes [4]. Early diagnosis of hepatosteatosis is essential to prevent NAFLD. NAFLD is a chronic liver disease that affects more than a quarter of the general population and is associated with obesity and metabolic syndrome [4]. A study reported that the development of cirrhosis and fibrosis is more common in people with NAFLD and CHB coexistence and even increases the risk of HCC [29]. This reveals the importance of hepatosteatosis in CHB.

Hepatosteatosis in HCV infection is associated with viral and metabolic factors [30]. It is known that hepatosteatosis seen in HBV infection is mainly affected by metabolic factors rather than viral factors [9]. Machado et al. examined 4100 patients with HBV infection in their meta-analysis of 21 studies. It was reported that hepatosteatosis is more common in CHB patients than in the average population [31]. In many studies around the world, the prevalence of hepatosteatosis in CHB patients varies greatly. Machado et al., in their meta-analysis of 21 studies, examined 4100 patients with HBV infection and reported that hepatosteatosis is more common in these patients than in the normal population [31]. In many studies around the world, the prevalence of hepatosteatosis in CHB patients varies greatly. While the rate of hepatosteatosis was 29.6% in the study of Machado et al., 38.9% in the study of Chen et al., it was found to be 40.9% in our study [24, 31].

Hepatosteatosis is affected by many factors such as race, genetics, diet, and sports habits. The similarities or differences in the results may be due to the fact that hepatosteatosis is related to demographic and metabolic characteristics instead of viral factors. In a study conducted in China, which did not include people with DM, dyslipidemia, and chronic liver disease that may predispose to metabolic disease, hepatosteatosis was found to be more common in people with a TyG index of 8.5 [28]. Fan et al. reported that the TG/HDL-C ratio is higher in people with hepatosteatosis and can be used as a marker showing NAFLD in their study on patients admitted to the hospital for health check-up [16]. Zheng et al., on the other hand, stated that insulin resistance is more common in CHB patients with hepatosteatosis [32]. Studies have shown that people with chronic hepatitis B and hepatosteatosis coexistence and those with high TyG index and TG/HDL-C ratio should be careful about metabolic diseases.

Hepatosteatosis in HCV infection is associated with viral and metabolic factors [30]. It is known that hepatosteatosis seen in HBV infection is affected by metabolic factors rather than viral factors [9]. Machado et al. examined 4100 patients with HBV infection in their meta-analysis of 21 studies, and it was reported that hepatosteatosis is more common in these patients than in the normal population [31]. In many studies around the world, the prevalence of hepatosteatosis in CHB patients varies greatly. While the rate of hepatosteatosis was 29.6% in the study of Machado et al. and 38.9% in Chen et al., it was found to be 40.9% in our study [24, 31]. Hepatosteatosis is affected by many factors such as race, genetics, diet, and sports habits. The similarities or differences in the results may be because hepatosteatosis is more related to demographic and metabolic characteristics than viral factors. In a study conducted in China, which did not include people with DM, dyslipidemia, and chronic liver disease that may predispose them to metabolic disease, hepatosteatosis was more common in people with a TyG index of 8.5 and above [28]. Fan et al. reported that the TG/HDL-C ratio is higher in people with hepatosteatosis and can be used as a marker showing NAFLD in their study on patients admitted to the hospital for health check-ups [16].

Zheng et al., on the other hand, stated that insulin resistance is more common in CHB patients with hepatosteatosis [32]. Studies have shown that people with chronic hepatitis B and hepatosteatosis coexistence and those with high TyG index and TG/HDL-C ratio should be careful about metabolic diseases.

Our study is a single-center study, and genotyping of HBV was not performed. Nutritional habits, daily calorie intake, and physical activities of the patients were not analyzed. In addition, the hepatosteatosis results of the patients were evaluated only with hepatobiliary USG. Since there is no indication for routine liver biopsy and it is an invasive procedure, liver biopsy was not performed in patients who did not receive antiviral therapy. Although only patients with minimal hepatic necro-inflammatory activity and low fibrosis levels were included in the study and those with chronic liver disease were excluded, the TG, glucose, and HDL-C values used in the calculation of the TyG index and TG/HDL-C ratio are not only related to insulin resistance but also liver disease, known to be affected by its function. Different states of glucose metabolism in the two groups were not distinguished, and the differences in the TyG index and TG/HDL-C ratio between different glucose metabolism statuses groups were not compared.

It has been determined that the TyG index and the TG/HDL-C ratio are practical alternatives for detecting insulin resistance. Still, studies examining factors such as insulin resistance in patients with HBeAg negative chronic HBV infection using the TyG index and TG/HDL-C ratio are not common in the literature. While insulin resistance is not emphasized in daily practice, HOMA-IR or other insulin measurements are more widely used [6]. The increase in studies evaluating the TyG index and TG/HDL-C ratio in these patients may increase the use of these indices in daily practice.

## Conclusion

The findings of the study support that the examination of the TyG index and TG/HDL-C in HBeAg negative chronic HBV infection patients is helpful in the diagnosis of insulin resistance and hepatosteatosis. Following the patients regarding the risk of metabolic disease development will help prevent complications related to these diseases.

**Conflicts of interest:** The authors declare no conflicts of interest.

**Funding:** The authors received no specific funding for this work.
